# Socioeconomic inequalities in childhood and adolescent body-mass index, weight, and height from 1953 to 2015: an analysis of four longitudinal, observational, British birth cohort studies

**DOI:** 10.1016/S2468-2667(18)30045-8

**Published:** 2018-03-21

**Authors:** David Bann, William Johnson, Leah Li, Diana Kuh, Rebecca Hardy

**Affiliations:** a Centre for Longitudinal Studies, University College London (UCL) Institute of Education, UCL, London, UK; b Population, Policy and Practice, UCL Great Ormond Street Institute of Child Health, UCL, London, UK; c Medical Research Council Unit for Lifelong Health and Ageing at UCL, UCL, London, UK; d School of Sport, Exercise and Health Sciences, Loughborough University, Loughborough, UK

## Abstract

**Background:**

Socioeconomic inequalities in childhood body-mass index (BMI) have been documented in high-income countries; however, uncertainty exists with regard to how they have changed over time, how inequalities in the composite parts (ie, weight and height) of BMI have changed, and whether inequalities differ in magnitude across the outcome distribution. Therefore, we aimed to investigate how socioeconomic inequalities in childhood and adolescent weight, height, and BMI have changed over time in Britain.

**Methods:**

We used data from four British longitudinal, observational, birth cohort studies: the 1946 Medical Research Council National Survey of Health and Development (1946 NSHD), 1958 National Child Development Study (1958 NCDS), 1970 British Cohort Study (1970 BCS), and 2001 Millennium Cohort Study (2001 MCS). BMI (kg/m^2^) was derived in each study from measured weight and height. Childhood socioeconomic position was indicated by the father's occupational social class, measured at the ages of 10–11 years. We examined associations between childhood socioeconomic position and anthropometric outcomes at age 7 years, 11 years, and 15 years to assess socioeconomic inequalities in each cohort using gender-adjusted linear regression models. We also used multilevel models to examine whether these inequalities widened or narrowed from childhood to adolescence, and quantile regression was used to examine whether the magnitude of inequalities differed across the outcome distribution.

**Findings:**

In England, Scotland, and Wales, 5362 singleton births were enrolled in 1946, 17 202 in 1958, 17 290 in 1970, and 16 404 in 2001. Low socioeconomic position was associated with lower weight at childhood and adolescent in the earlier-born cohorts (1946–70), but with higher weight in the 2001 MCS cohort. Weight disparities became larger from childhood to adolescence in the 2001 MCS but not the earlier-born cohorts (p_interaction_=0·001). Low socioeconomic position was also associated with shorter height in all cohorts, yet the absolute magnitude of this difference narrowed across generations. These disparities widened with age in the 2001 MCS (p_interaction_=0·002) but not in the earlier-born cohorts. There was little inequality in childhood BMI in the 1946–70 cohorts, whereas inequalities were present in the 2001 cohort and widened from childhood to adolescence in the 1958–2001 cohorts (p_interaction_<0·05 in the later three cohorts but not the 1946 NSHD). BMI and weight disparities were larger in the 2001 cohort than in the earlier-born cohorts, and systematically larger at higher quantiles—eg, in the 2001 MCS at age 11 years, a difference of 0·98 kg/m^2^ (95% CI 0·63–1·33) in the 50th BMI percentile and 2·54 kg/m^2^ (1·85–3·22) difference at the 90th BMI percentile were observed.

**Interpretation:**

Over the studied period (1953–2015), socioeconomic-associated inequalities in weight reversed and those in height narrowed, whereas differences in BMI and obesity emerged and widened. These substantial changes highlight the impact of societal changes on child and adolescent growth and the insufficiency of previous policies in preventing obesity and its socioeconomic inequality. As such, new and effective policies are required to reduce BMI inequalities in childhood and adolescence.

**Funding:**

UK Economic and Social Research Council, Medical Research Council, and Academy of Medical Sciences/the Wellcome Trust.

## Introduction

Reducing socioeconomic inequalities in childhood and adolescent obesity is an important public policy goal because of its multiple long-term adverse health consequences.^1^, ^2^ A priority is to understand how health inequalities have changed over time and understand whether policy goals of health inequality reduction are being met.^3^ Although socioeconomic inequalities in childhood overweight have been documented in high-income countries,^4^ it remains unclear how these inequalities have changed across generations;^5^ interpretation of existent data is limited by the short timespan of previous investigations (eg, 5–10 years), gaps in timespans investigated, and methodological differences across studies.

**Research in context**

**Evidence before this study**

We searched PubMed for articles and reviews published between Jan 1, 1960, and Oct 9, 2017, using the search terms “body mass index” OR “obesity” AND “socioeconomic” OR “inequality” OR “disparity”. We screened published articles by title and abstract to identify relevant studies of how socioeconomic inequalities in body-mass index (BMI) or obesity risk had changed across time. The studies cited in this report are not an exhaustive list of existing research. Published systematic reviews have found many studies that document the existence of socioeconomic inequalities in childhood and adolescence BMI or obesity risk in high-income countries. However, evidence for how these inequalities have changed over time is typically short term and cross-sectional in nature, does not examine the composite parts of BMI (ie, weight and height), and does not examine whether inequalities differ in magnitude across the outcome distribution. To inform public policy—and specific concerns regarding the adverse long-term consequences of childhood obesity and its socioeconomic inequality—robust and nationally representative evidence is required to examine how inequalities have changed in response to shifting policy and societal factors.

**Added value of this study**

We used four British historic longitudinal studies to examine trends in socioeconomic inequalities in BMI from 1953 to 2015. This study provides added value by enabling a long-run investigation of socioeconomic inequalities in BMI, and more recent data than previously available. Most existing evidence is cross-sectional in nature; however, we used longitudinal data and found that absolute socioeconomic inequalities in BMI widened from childhood to adolescence. We also examined the different components of BMI that yielded new policy-relevant evidence; absolute height inequalities have narrowed in subsequent generations whereas weight inequalities have reversed (ie, changed direction). Finally, we examine how inequalities in these outcomes differ across the outcome distribution using quantile regression, in which we observed that socioeconomic inequalities in BMI were found at the median but were systematically larger at higher BMI quantiles than at lower quantiles.

**Implications of all available evidence**

The emergence and widening of socioeconomic inequalities in BMI in children and adolescents up to 2015 suggests a renewed need for effective policies to reduce obesity and its socioeconomic inequality in current and future generations; previous policies have not been adequate, and existing policies are unlikely to be either. Without effective intervention, socioeconomic inequalities in BMI are anticipated to widen further throughout adulthood and disproportionately affect those who have higher BMI, leading to decades of adverse health and economic consequences.

Additionally, several important aspects of the nature of socioeconomic inequalities in BMI remain poorly understood; in particular, the extent to which socioeconomic inequalities have changed across the composite parts of BMI (ie, weight and height). Because lower socioeconomic position has been associated with shorter childhood height,^6^ changes in BMI might be attributable to changes in weight or height, or both. Understanding both components separately is important because of the association between shorter height in childhood and increased premature mortality risk,^7^ and the association between shorter height in adulthood and increased adult cardiovascular disease risk.^8^ Socioeconomic inequalities in these constituent parts might have changed in different ways over time. For example, improvements in population micronutrient intake and reductions in early life infections might have occurred, as suggested by secular trends towards taller childhood height from 1957 to 2012,^9^ and suggestive evidence for reduced prevalence of stunting in Britain.^7^ These changes might have also led to narrower height inequalities in recent decades, yet increases in total calorie consumption associated with the recent obesity burden might have led to the emergence and widening of weight and thus BMI inequalities from the 1980s onwards. BMI inequalities might also lead to narrower height inequalities, because obesity can increase the pace of pubertal development.^10^ Additionally, existing evidence for how BMI inequalities have changed over time is typically from repeated cross-sectional studies, limiting the understanding of the ages at which inequalities emerge or widen. Finally, the effect of socioeconomic inequalities on the population distribution of BMI, height, and weight is not well understood. A trend towards an increasing BMI across time has been observed,^9^, ^11^ and this increase could be disproportionately attributable to people in disadvantaged socioeconomic groups.^12^

Therefore in this study, we aimed to examine trends in socioeconomic inequalities in BMI, weight, and height across childhood to adolescence using data from four British birth cohort studies, enabling a long-run comparison from 1953 to 2015. We hypothesised that socioeconomically disadvantaged groups had lower bodyweight and shorter height than socioeconomically advantaged groups born in the mid-to-late 20th century; among those born in the early 21st century, we hypothesised that differences in bodyweight would have reversed and that height differences would be narrower.

## Methods

### Study design and samples

We used data from four longitudinal, observational, British birth cohort studies. These cohorts have been previously described in detail elsewhere,^9^, ^13^, ^14^ and they were designed to be nationally representative when initiated in 1946 (MRC National Survey of Health and Development [1946 NSHD]), 1958 (National Child Development Study [1958 NCDS]), 1970 (British Cohort Study [1970 BCS]), and 2001 (Millennium Cohort Study [2001 MCS]). We categorised individuals born in the mid-to-late 20th century (ie, the 1946 NSHD, 1958 NCDS, and 1970 BCS) as the earlier-born cohorts, and those born in the early 21st century (ie, the 2001 MCS) as the later-born cohort.

To aid comparability, we restricted the analyses to singleton births in England, Scotland, and Wales in 1946, 1958, 1970, and 2001; however, only singletons were sampled in the 1946 NSHD.

### Weight, height, and BMI measurements

As described elsewhere,^9^ BMI (kg/m^2^) was derived and harmonised in each study from measured weight and height. These three outcomes were obtained at the following ages: 7 years, 11 years, and 15 years in the 1946 NSHD; 7 years, 11 years, and 16 years in the 1958 NCDS; 10 years and 16 years in the 1970 BCS; and 7 years, 11 years, and 14 years in the 2001 MCS.

### Ascertainment of socioeconomic position

Childhood socioeconomic position was indicated by the father's (occupational) social class, reported at the ages of 10–11 years. To assist cross-cohort comparability, the Registrar-General's Social Classes was used to classify social class by occupational group: I (professional), II (managerial and technical), IIIN (skilled non-manual), IIIM (skilled manual), IV (partly skilled), and V (unskilled).^15^ The 1990 classification schema was used for all cohorts except for the 1946 NSHD, for which the 1970 version was used because of the absence of a conversion schema. Additionally, the 1970 classification schema was used if historic source data were not retrievable. Those in the armed forces or who were unemployed were not assigned a social class. We used mother-figure occupational class when no father-figure was present in the household or for which no valid father-figure occupational class data were available in the 2001 MCS, because of recent increases in this type of family composition.

### Statistical analysis

To account for differences in the exact age of measurement across cohorts, we calculated age-centred BMI, weight, and height values at ages 7 years, 11 years, and 15 years using predictions from cohort-specific linear regression models of age regressed on these outcomes (ie, BMI, weight, and height). We found little evidence for gender differences in associations between social class and these outcomes (gender × socioeconomic position interactions); as such, we conducted gender-pooled and gender-adjusted models, which were consistent with previous analyses that used the 2001 MCS.^16^, ^17^, ^18^ To provide single quantifications of inequalities, we converted social class to ridit scores (ranging from 0 to 1) calculated separately in each cohort. The socioeconomic position coefficient in linear regression—the slope index of inequality—is interpreted as the estimated absolute (mean) difference (absolute inequality) in outcome between the lowest and highest socioeconomic position. This method enables comparisons even when the proportion of participants differs in each socioeconomic position category across cohorts. We also used multilevel models to examine whether absolute inequalities systematically changed by age; age × ridit score interaction terms were included in models with outcome measurements (level 1) nested within individuals (level 2). Additionally, we specified a random intercept and random slope, as well as modelled age as a linear term. In the 1970 BCS, we did not specify random effects because the maximal number of observations was two (participants were aged 10 years and 16 years).

We used conditional quantile regression to examine associations between social class ridit scores and outcomes at specific quantiles of distributions of BMI, weight, and height. We obtained estimates and plotted them at the following quantiles: fifth, tenth, 25th, 50th (median), 75th, 90th, and 95th. Additionally, we used multinomial regression to examine associations between social class ridit scores and International Obesity Task Force BMI thresholds; these thresholds are age-specific cutpoints designed to correspond to adult BMI cutpoints of thinness (<18·5 kg/m^2^), normal weight (18·5 to <25 kg/m^2^), overweight (25 to <30 kg/m^2^), and obesity (≥30 kg/m^2^).^19^ We tested the associations between socioeconomic position and this categorical outcome on both the absolute scale (difference in predicted probability of each outcome—ie, risk difference) and relative scale (relative risk ratio, with normal BMI as the reference).

We repeated all analyses using maternal education attainment (ascertained at age 6 years in the 1946 NSHD, at birth in the 1958 NCDS and 1970 BCS, and at 9 months of age in the 2001 MCS) instead of the father's social class, which has previously been shown to be related to anthropometric outcomes in the included cohorts (eg, the 2001 MCS^16^, ^17^). These analyses might also provide a means of triangulation for causal inference, since consistency of findings based on both maternal education and paternal social class indicators suggest that findings are not solely explained by confounding factors acting on one parent figure. We used two measures: a binary indicator of whether the mother had left education at the mandatory leaving age (14 years from 1918, 15 years from 1944, and 16 years from 1972), and the age the mother left full-time education (in 10-year age groups from <13 years to ≥23 years, measured at age 16 years in the 1958 NCDS). We modelled both of these measures as ridit scores to aid comparability. To examine whether differences in ethnic composition affected results of cross-cohort comparisons, we repeated linear regression and quantile analyses restricted to white participants only.

We weighted the analyses where appropriate to account for the survey design of the 1946 NSHD and 2001 MCS, whereas analyses using the 1970 BCS and 1958 NCDS were not weighted because no subgroups were over or under sampled. We did all analyses using Stata (version 15.0).

### Data sharing

The harmonised BMI dataset is freely available to download at the UK Data Archive. Additionally, all original datasets from the 1958 NCDS, 1970 BCS, and 2001 MCS are freely available to download at the UK Data Archive. Additional data from the 1946 NSHD are made freely available to researchers who submit data requests to the NSHD Data Archive.

### Role of the funding source

The funders of the study had no role in study design, data collection, data analysis, data interpretation, or writing of the report. The corresponding author had full access to all the data in the study and had final responsibility for the decision to submit for publication.

## Results

In England, Scotland, and Wales, 5362 singleton births were enrolled in 1946, 17 202 in 1958, 17 290 in 1970, and 16 404 in 2001. All participants in the 1946 NSHD were white, as were 14 407 (98·7%) of 14 603 in the 1958 NCDS (2599 had missing ethnicity data), and 13 671 (95·2%) of 14 354 in the 1970 BCS (2936 had missing ethnicity data). In the 2001 MCS, only 13 208 (80·5%) of 16 404 were white. The table summarises the sample sizes for analyses by age group in each cohort. In 2318 participants in the 1958 NCDS, the 1970 classification schema was used because of irretrievable historic source data (Spearman's correlation coefficient between social class derived by 1970 and 1990 schema *r*=0·77; p<0·0001). The proportion of participants with no father figure at the age of 10–11 years was low in the early cohorts: unmarried women were not sampled in the 1946 NSHD and less than 3·6% of participants had no father figure at the age of 10–11 years in both the 1958 NCDS and 1970 BCS. No valid father-figure occupational class data were available for 2430 participants in the 2001 MCS; low mother-figure class was associated in expected directions with low maternal education attainment (p<0·0001) and low father's social class (p<0·0001).

**Table:** Averages and socioeconomic differences in BMI, weight, and height during childhood to adolescence

	**Year**	**n**	**BMI (kg/m^2^)**			**Weight (kg)**			**Height (cm)**		
			Mean (SD)	Median (IQR)	SEP difference, SII (95% CI)	Mean (SD)	Median (IQR)	SEP difference, SII (95% CI)	Mean (SD)	Median (IQR)	SEP difference, SII (95% CI)
**Children aged 7 years**											
1946 NSHD	1953	3510	15·8 (1·5)	15·6 (14·9–16·6)	0·0 (−0·2 to 0·2)	22·5 (3·1)	22·2 (20·4–24·2)	–1·4 (−1·9 to −0·9)	119·3 (5·6)	119·4 (116·4–123·2)	–3·9 (−4·6 to −3·1)
1958 NCDS	1965	10 650	15·7 (1·8)	15·5 (14·7–16·5)	0·1 (−0·1 to 0·2)	22·7 (3·7)	22·2 (20·3–24·4)	–1·1 (−1·3 to −0·8)	120·8 (5·8)	120·6 (117·3–124·1)	–3·0 (−3·4 to −2·6)
2001 MCS	2008	8340	16·4 (2·2)	16·0 (15·0–17·2)	0·5 (0·3 to 0·7)	24·5 (4·6)	23·7 (21·4–26·6)	0·3 (−0·1 to 0·8)	122·6 (5·3)	122·4 (119·0–126·1)	–1·2 (−1·7 to −0·8)
**Children and adolescents aged 11 years**											
1946 NSHD	1957	3629	17·4 (2·4)	17·0 (15·9–18·4)	–0·1 (−0·4 to 0·3)	34·9 (6·5)	33·7 (30·5–37·8)	–2·0 (−3·0 to −1·1)	141·0 (6·9)	140·6 (135·9–145·7)	–4·1 (−5·1 to −3·2)
1958 NCDS	1969	11 193	17·3 (2·6)	16·7 (15·6–18·3)	0·0 (−0·2 to 0·1)	35·1 (7·3)	33·6 (30·1–38·6)	–1·8 (−2·3 to −1·3)	142·3 (7·1)	142·2 (137·6–146·9)	–3·5 (−3·9 to −3·0)
1970 BCS	1980	11 231	17·4 (2·1)	17·1 (16·0–18·4)	0·1 (0·0 to 0·3)	35·8 (5·3)	35·0 (32·0–38·7)	–1·0 (−1·3 to −0·6)	142·2 (6·4)	142·0 (137·9–146·3)	–2·7 (−3·1 to −2·3)
2001 MCS	2012	8820	18·9 (3·4)	18·2 (16·5–20·7)	1·3 (0·9 to 1·6)	40·5 (9·4)	38·9 (33·8–45·4)	2·1 (1·2 to 2·9)	145·7 (6·9)	145·5 (141·2–150·3)	–1·2 (−1·7 to −0·6)
**Children and adolescents aged 15 years**											
1946 NSHD	1961	3262	20·4 (2·8)	20·0 (18·5–21·7)	0·2 (−0·2 to 0·7)	53·3 (9·2)	52·5 (47·0–58·2)	–1·9 (−3·3 to −0·5)	162·2 (8·0)	162·2 (157·1–167·3)	–4·0 (−5·2 to −2·9)
1958 NCDS	1973	8824	20·2 (2·9)	19·7 (18·3 −21·5)	0·4 (0·1 to 0·6)	53·7 (9·7)	52·5 (47·3–58·8)	–1·3 (−2·1 to −0·6)	161·7 (8·5)	161·3 (155·6–167·2)	–3·3 (−3·9 to −2·8)
1970 BCS	1986	6649	20·2 (3·1)	19·7 (18·2–21·6)	0·6 (0·3 to 0·9)	53·5 (10·1)	52·3 (46·8–58·9)	–0·5 (−1·3 to 0·4)	161·4 (9·4)	161·3 (154·7–167·7)	–3·0 (−3·6 to −2·3)
2001 MCS	2015	7393	21·7 (3·9)	20·9 (19·0–23·5)	1·4 (1·0 to 1·8)	60·9 (12·1)	59·2 (52·8–70·0)	2·4 (1·2 to 3·6)	168·9 (7·9)	168·5 (163·3–174·0)	–1·7 (−2·4 to −1·0)

Means and SII are gender-adjusted, and outcomes were age-centred at 7 years, 11 years, and 15 years. BMI=body-mass index. NSHD=Medical Research Council National Survey of Health and Development. NCDS=National Child Development Study. BCS=British Cohort Study. MCS=Millennium Cohort Study. SEP=socioeconomic position (or social class characterised by father's occupation). SII=slope index of inequality.

Some descriptive trends were observed when comparing the 2001 MCS with the 1946 NSHD, 1958 NCDS, and 1970 BCS: BMI, weight, and height values were higher in the 2001 MCS than in the earlier-born cohorts (table; appendix pp 1, 2). The composition of socioeconomic position also differed across the cohorts: proportions in the managerial to technical occupational groups were highest in the 2001 MCS, and skilled manual occupations were lowest in this cohort (appendix p 3). Post compulsory education attendance was higher in the 2001 MCS cohort than in the earlier-born cohorts. Missing socioeconomic position and missing BMI data were more frequent in the 2001 MCS than in the earlier-born cohorts, whereas missing maternal education was more frequent in the 1946 NSHD than in the 2001 MCS (appendix p 3). For example, 1425 (26·6%) of the 5362 sampled at birth in the 1946 NSHD did not have their BMI measured at age 11 years compared with 5408 (33·0%) of the 16 404 in the 2001 MCS. Reasons for these missing measurements included death, emigration, and loss to follow-up. Missing data at 14–16 years were in most instances more frequent in participants of low socioeconomic position at 10–11 years and with high preceding BMI (appendix p 4).

In the earlier-born cohorts, lower socioeconomic position at all ages was associated with lower weight, whereas only in the 2001 MCS was lower social class associated with higher weight (table). These differences did not systematically differ by age in the 1946 NSHD, 1958 NCDS, or 1970 BCS cohorts. However, in the 2001 MCS cohort, weight disparities became larger from childhood to adolescence (p_interaction_=0·001 for social class × age; appendix p 5).

**Figure 1:**
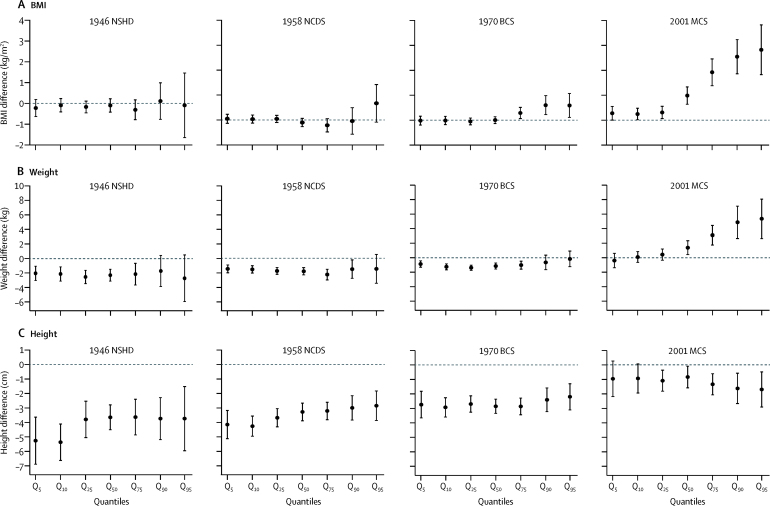
Estimated differences in BMI (A), weight (B), and height (C) in children aged 11 years in the lowest social class compared with the highest social class* (slope index of inequality) Data are quantile regression estimates at different quantiles of the outcome distribution. Error bars are 95% CI. Coefficients are interpreted analogously to linear regression—eg, Q_50_ shows the median difference in BMI comparing the lowest with highest social class. BMI=body-mass index. NSHD=MRC National Survey of Health and Development. NCDS=National Child Development Study. BCS=British Cohort Study. MCS=Millennium Cohort Study. *Social class characterised by father's occupation.

**Figure 2:**
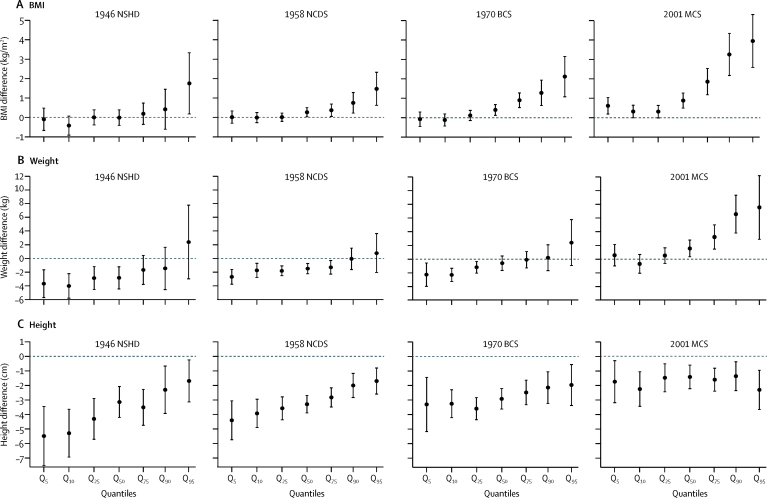
Estimated differences in BMI (A), weight (B), and height (C) in children aged 15 years in the lowest social class compared with the highest social class* (slope index of inequality) Data are quantile regression estimates at different quantiles of the outcome distribution. Error bars are 95% CI. Coefficients are interpreted analogously to linear regression—eg, Q_50_ shows the median difference in BMI comparing the lowest with highest social class. BMI=body-mass index. NSHD=MRC National Survey of Health and Development. NCDS=National Child Development Study. BCS=British Cohort Study. MCS=Millennium Cohort Study. *Social class characterised by father's occupation.

In the 2001 MCS, inequalities in weight were present at the median, and became increasingly larger at higher quantiles (figure 1B). For example when comparing lowest with highest social class at age 11 years, there was a difference of 1·40 kg (95% CI 0·44–2·35) at the 50th weight percentile whereas a difference of 4·88 kg (2·66–7·10) was observed at the 90th weight percentile. Inequalities in weight were comparatively similar across quantiles in the earlier-born cohorts. These findings were similar at age 15 years (figure 2B).

In all cohorts, lower socioeconomic position was associated with shorter height, yet the absolute magnitude of this difference narrowed in each subsequent cohort (table). These associations became more negative (ie, height disparities widened in absolute terms) with age in the 2001 MCS (p_interaction_=0·002) but not in the earlier-born cohorts (p_interaction_=1·00 in the 1946 NSHD, p_interaction_=0·29 in the 1958 NCDS, and p_interaction_=0·51 in the 1970 BCS for social class × age; appendix p 5).

In all cohorts, inequalities in height did not appear to systematically differ across the quantiles for those aged 11 years or 15 years (Figure 1, Figure 2).

**Figure 3:**
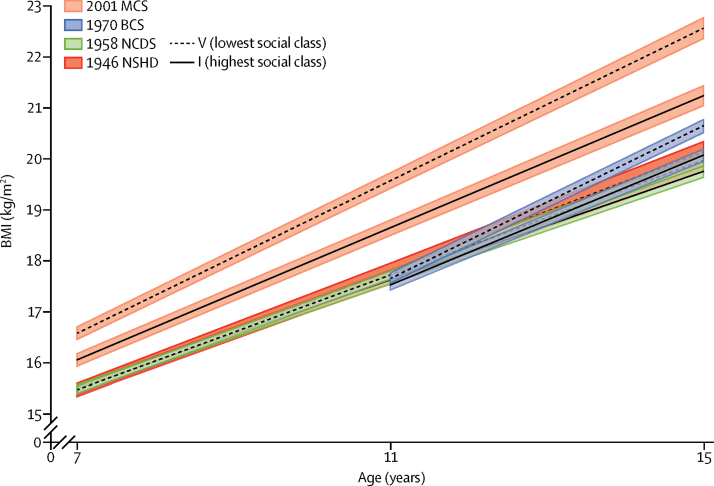
BMI across childhood to adolescence by social class* in four British birth cohort studies Lines are estimated BMI and widths of the shaded area are 95% CIs at each age among women, estimated with multilevel general linear regression models (the appendix shows the full model estimates). BMI=body-mass index. NSHD=MRC National Survey of Health and Development. NCDS=National Child Development Study. BCS=British Cohort Study. MCS=Millennium Cohort Study. *Social class characterised by father's occupation.

There was little evidence for socioeconomic inequality in mean BMI at age 7 years or 11 years in the 1946 NSHD, 1958 NCDS, or 1970 BCS; however, inequalities were present in the 2001 MCS at ages 7 years and 11 years (table). Mean BMI differences by socioeconomic position were present in all cohorts except the 1946 NSHD at age 15 years, and this difference remained substantially larger in the 2001 MCS. Inequalities generally widened with age from 7 years or 10 years to 15 years (social class × age interaction terms were positive in all cohorts: p_interaction_=0·159 in the 1946 NSHD, p_interaction_=0·004 in the 1958 NCDS, p_interaction_=0·001 in the 1970 BCS, and p_interaction_<0·001 in the 2001 MCS; figure 3; appendix p 5).

Quantile regression analyses suggested that in the 2001 MCS at age 11 years, inequalities in BMI were present at the median and increasingly became larger at higher quantiles (eg, when comparing lowest with highest social class, a difference of 0·98 kg/m^2^ [95% CI 0·63–1·33] at the 50th BMI percentile and a difference of 2·54 kg/m^2^ [1·85–3·22] at the 90th percentile; figure 1A). Such patterns were also found in all cohorts at age 15 years (figure 2A), but the magnitude of inequalities was larger in the 2001 MCS than in the earlier-born cohorts. These findings were consistent with visual inspection of BMI distributions in different socio-economic groups, suggesting more skewness in BMI distributions among lower socioeconomic groups, and results from multinomial regression analyses, which suggested that lower social class was associated with increased absolute risk of overweight or obesity at age 15 years in cohorts born in 1958–2001, but no increased absolute risk of thinness (appendix p 6). When examined in the absolute scale (differences in predicted probabilities of each BMI category), inequalities in overweight or obesity were higher in the 2001 MCS than in the earlier-born cohorts, which was consistent with the main findings (appendix p 6). These patterns of results were similar when the UK 1990 growth reference thresholds were used (data not shown).

Results were similar when maternal education attainment was used as an alternative indicator of socioeconomic position (appendix pp 1, 2, 7–9). Findings were similar when restricted to only white participants (eg, quantile regression estimates for those in the 2001 MCS aged 11 years shown in the appendix [p 10]).

## Discussion

In four national British birth cohorts with data spanning from 1953 to 2015, socioeconomic inequalities in weight reversed: lower socioeconomic position was associated with lower weights in the 1946, 1958, and 1970 cohorts but in the 2001 cohort it was associated with higher weight; whereas lower socioeconomic position was associated with shorter height in all cohorts but the absolute magnitude of this difference narrowed in each subsequent cohort. The magnitude of absolute inequalities in BMI differed in each cohort as a result, and was larger and apparent earlier in childhood in the 2001 MCS than in the earlier-born cohorts (ie, the 1946 NSHD, 1958 NCDS, and 1970 BCS). BMI differences widened from childhood to adolescence in all cohorts except the 1946 NSHD. These findings were consistent when using both father's social class and maternal education as indicators of socioeconomic position.

Our findings are consistent with existing cross-sectional evidence from the UK, suggesting that relative inequalities in obesity or BMI have increased in recent decades. This trend was observed in the analysis of obesity inequalities from 1997 to 2005 among children aged 5–10 years in the Health Survey for England,^20^ in BMI inequalities among those aged 4–5 years and 10–11 years in 2007–08 and 2011–12 in the National Child Measurement Programme,^12^ and in obesity inequalities among those aged 10–11 years in the 1970 BCS and 2001 MCS (1980 compared with 2011).^21^ Here, we show that BMI inequalities have persisted to 2015, and that in multiple generations absolute and relative BMI inequalities widened with age from childhood to adolescence. We also show, as suggested in recent cross-sectional data,^12^ that BMI inequalities were larger at the higher end of the BMI distribution. These findings are consistent with, and might partially explain, the observed positive skew of the population BMI distribution in later-born cohorts.^9^, ^11^

Our finding that height inequalities have narrowed is consistent with suggestive evidence from a study comparing height inequalities among the 1958 NCDS and their children.^22^ Narrowing of height differences has also been reported in other countries that have had substantial economic and nutritional changes.^23^

Considerable changes took place in the period investigated (1953–2015) in Britain, including changes to several factors that might have ultimately influenced diet and physical activity, which are the plausible yet equivocal mediators of BMI and height inequalities.^24^, ^25^, ^26^ Diets in both the prenatal and postnatal periods are likely to contribute to BMI and height inequalities,^27^ and British diets have changed considerably. World War 2-related food rationing continued up to 1954 in the UK; compared with population diet in the 1990s, rationing-based diets were characterised by higher consumption of vegetables, lower consumption of sugar and soft drinks, and higher consumption of fat as a proportion of energy intake.^28^ Despite rationing, socioeconomic inequalities in diet were documented at age 4 years in the 1946 NSHD, in which children of lower socioeconomic groups consumed fewer total calories as well as fruit and vegetables, and thus fewer micronutrients such as zinc and potassium than those of higher socioeconomic position.^29^ These differences in diet might underlie the association between low socioeconomic position and both lower weight and shorter height in the 1946 NSHD.^30^ From 1953 to 2015, inequalities in micronutrient intake might have reduced leading to narrower height inequalities;^31^ inequalities in total calorie consumptions are likely to have reversed over time leading to those of lower socioeconomic position having higher weight, BMI, and obesity risk.

Income and wealth inequality have increased since the 1970s,^32^ and some evidence suggests that the price of healthy food items has increased in recent decades.^33^ These changes might also have contributed to the emergence and widening of BMI inequalities. Among later-born cohorts, a study found inequalities in childhood exercise participation and sedentary behaviour,^18^ but such inequalities might be weaker or not present in earlier-born cohorts that predate the routine collection of such data. Increases in BMI among adults from the 1980s onwards,^9^ combined with the persistence of adult BMI inequalities,^14^ might have indirectly contributed to increases in BMI and weight inequalities among children, since in all cohorts higher parental BMI was associated with higher offspring BMI.^34^

Additionally, we observed that inequalities in BMI were larger at the higher end of the distribution than at the midpoint (median) or lower end of the distribution. These findings could be explained by unmeasured modifiers that acted to increase the magnitude of BMI inequality. For example, individuals who were more susceptible to higher BMI (for environmental or genetic reasons, or both) might have been more susceptible to the adverse effects of socioeconomic disadvantage,^35^ leading to inequalities in BMI being larger at the higher end of the BMI distribution, as observed in our quantile regression results.

Our study had several strengths that included the use of four national birth cohort studies, enabling investigation of long-term trends in BMI, weight, and height. Inferences regarding cross-cohort comparisons were strengthened by the use of harmonised socioeconomic and anthropometric data, comparable sample restrictions, and analyses that accounted for the differing sampling design in each cohort. However, although the study samples used were generally large, they were underpowered to evaluate socioeconomic inequalities in thinness,^16^ or differences across subgroups of race or ethnicity, in whom both past and future trends in inequalities might differ. Additionally, the 30-year gap from 1970 to 2001, in which no national birth cohorts were conducted, prevents the investigation of such cohorts born in this period.

Despite the use of national data, our data potentially provide inexact approximations of existing health inequalities in Britain. Inequalities in fat might have been underestimated because BMI consists of, but does not distinguish, fat and lean mass; and because lower socioeconomic position has been associated with higher fat yet not associated with lean mass in children.^36^ Our analyses were designed to maximise cross-cohort comparability, including the use of harmonised socioeconomic position data for the father's social class and maternal education. Our findings were consistent across both of these indicators. This consistent finding was encouraging because each indicator had differing strengths and weaknesses. Compared with maternal education, social class had more missing data but was measured at a similar age and arguably contained more information across the socioeconomic distribution in each cohort. Consistency of findings also suggests that the results are not solely explained by confounding due to factors affecting one particular parent (eg, maternal health); however, the potential of confounding due to other shared factors cannot be ruled out. Although the same social class categories were used in all cohorts, the use of the 1970 schema to derive this categorisation in the 1946 NSHD but not the other cohorts could theoretically affect comparisons. However, we expect that this difference was unlikely to have a major effect on findings since the 1970 and 1990 versions were strongly correlated. The use of these indicators is potentially at the expense of obtaining the most informative estimates of inequality available in individual cohorts (eg, detailed parental education and household income data in the 2001 MCS). The use of slope indices of inequality aided comparisons of inequality by accounting for differences in the proportions of participants in the socioeconomic position categories in each cohort. However, as with all studies investigating trends in socioeconomic inequalities, changes in the selection into different socioeconomic groups might differ over time. In this scenario, even when the statistical estimates of inequality are comparable (as in the slope index of inequality), interpretation might not be.

Missing data, which might be due to death, emigration from Britain, dropout, and refusal to participate, might have also introduced bias into the inequality estimates. Attrition in longitudinal studies is generally greatest in those of lower socioeconomic position and higher BMI, and greater attrition of this type has been shown to lead to a reduction in the magnitude of observed health inequalities.^37^ Since this pattern of missing data was also found in the 2001 MCS but not the 1946 NSHD, we might have underestimated the increase in BMI inequalities over time, although accounting for missing data in the 2001 MCS has been reported to not substantially alter findings.^17^ Because of a teachers strike in 1986, missing anthropometric data were particularly substantial at age 16 years in the 1970 BCS, which might have primarily affected statistical power rather than biasing estimates since the cause of missing data was possibly unrelated to participant characteristics. Although our findings were similar when using multilevel models that enable those with incomplete information to be included in analyses (under the assumption of missing at random), as in all observational studies, we cannot rule out the possibility that missing data are non-ignorable and therefore might upwardly or downwardly bias inequality estimates.

Our results suggest that the total effect of previous policies has been insufficient in preventing the emergence and widening of BMI inequalities in childhood and adolescence from 1953 to 2015. In Britain, numerous policy initiatives have been created to tackle obesity since 1991, which have differed in their ambition, funding, implementation, and suitability for evaluation.^38^ Our results show that powerful influence of the obesogenic environment has disproportionately affected socioeconomically disadvantaged children from 1953 to 2015. Our results reinforce the need for new approaches, particularly given absolute increases in BMI inequality with age. Without effective intervention, these inequalities are anticipated to widen further throughout adulthood in the 2001 MCS and future cohorts,^14^ with considerable public health and economic implications.^39^, ^40^

Globally, the need to reduce childhood obesity prevalence and its socioeconomic inequality has been repeatedly noted, yet policy responses are often assessed as ineffectual or inappropriately focused on individual or family agency rather than upstream societal factors.^41^, ^42^ Current policies in the UK, include the so-called Change4Life, a social marketing campaign aimed at families and individuals; and a forthcoming tax on soft drinks (the Soft Drinks Industry Levy) that notably excludes other sugary drinks and food items. Although the empirical evidence for what could reduce population-level obesity and its inequality is scarce,^39^ committed cross-government action is required on legislative changes rather than voluntary suggestions, which might help reverse the obesogenic environment—eg, further legislative incentives to food manufacturers to reduce sugar and fat content in food and drinks, as well as the advertising of such foods to children and parents, while incentivising the sale of healthier alternatives.

Finally, our results of quantile regression analyses have potential policy implications. Because socioeconomic inequalities appear to disproportionately affect those of higher BMI, an additional effective means of reducing socioeconomic inequalities in BMI might be to target those of particularly high BMI. Alternatively, assuming a causal link, reducing socioeconomic inequalities in society might benefit population health by dis-proportionately lowering BMI in those with particularly high BMI values. By contrast, socioeconomic inequalities in height were similar across the distribution of height. The fact that socioeconomic inequalities in childhood and adolescent height have persisted up to 2015 suggests that new policies are required to reduce them. The narrowing of absolute height inequalities that we observed might have been favourable to public health. However, increases in BMI inequality are likely to have a greater adverse effect, because absolute risk of cardiovascular disease attributable to height is small compared with that to BMI, and taller stature might not always benefit health (eg, it is associated with increased risk of some types of cancers^43^).

In conclusion, between the late 20th and early 21st centuries, socioeconomic inequalities in weight reversed (ie, changed direction) and those in height narrowed, whereas inequalities in BMI and obesity emerged and widened. These substantial changes highlight the powerful impact of societal changes on child and adolescent growth and the insufficiency of previous policies in preventing obesity and its socioeconomic inequality. New and effective policies are required to reduce BMI inequalities in current and future children and adolescents. Without effective interventions, these inequalities are anticipated to widen further throughout adulthood.

For the **UK Data Archive** see http://www.data-archive.ac.uk

For the **NSHD Data Archive** see http://www.nshd.mrc.ac.uk/data.aspx
