# Supplementary material for: Socioeconomic inequalities in childhood and adolescent body-mass index, weight, and height from 1953 to 2015: an analysis of four longitudinal, observational, British birth cohort studies
Source: Lancet Public Health. 2018 Mar 21;3(4):e194–203. doi: 10.1016/S2468-2667(18)30045-8 (PMC5887082; doi:10.1016/S2468-2667(18)30045-8)
Supplement: Supplementary appendix [file mmc1.pdf]

THE LANCET
Public Health

Supplementary appendix

This appendix formed part of the original submission and has been peer reviewed. We post it as supplied by the authors.

Supplement to: Bann D, Johnson W, Li L, Kuh D, Hardy R. Socioeconomic inequalities in childhood and adolescent body-mass index, weight, and height from 1953 to 2015: an analysis of four longitudinal, observational, British birth cohort studies. *Lancet Public Health* 2018; published online March 20. http://dx.doi.org/10.1016/S2468-2667(18)30045-8.

Supplementary Table 1. Mean (SE) BMI, weight and height in childhood/adolescence by parental socioeconomic position

Body mass index		Father's social class						Maternal education	
Cohort	Age	I Professional	II	III Non-manual	III Manual	IV	V Unskilled	High	Low (compulsory)
1946 NSHD	7	15.9 (0.1)	15.9 (0.1)	15.6 (0.1)	15.8 (0.0)	15.8 (0.1)	15.9 (0.1)	15.7 (0.1)	15.8 (0.0)
	11	17.6 (0.2)	17.6 (0.1)	17.3 (0.1)	17.4 (0.1)	17.4 (0.1)	17.5 (0.2)	17.3 (0.1)	17.5 (0.1)
	15	20.1 (0.2)	20.5 (0.1)	20.0 (0.1)	20.3 (0.1)	20.6 (0.1)	20.5 (0.3)	20.2 (0.1)	20.5 (0.1)
1958 NCDS	7	15.7 (0.1)	15.8 (0.0)	15.6 (0.0)	15.8 (0.0)	15.7 (0.0)	15.8 (0.1)	15.7 (0.0)	15.7 (0.0)
	11	17.1 (0.1)	17.3 (0.1)	17.1 (0.1)	17.3 (0.0)	17.3 (0.1)	17.1 (0.1)	17.1 (0.0)	17.3 (0.0)
	16	19.8 (0.1)	20.1 (0.1)	20.0 (0.1)	20.3 (0.0)	20.3 (0.1)	20.1 (0.1)	20.0 (0.0)	20.3 (0.0)
1970 BCS	10	17.3 (0.1)	17.3 (0.0)	17.3 (0.1)	17.4 (0.0)	17.5 (0.1)	17.3 (0.1)	17.3 (0.0)	17.4 (0.0)
	16	19.9 (0.1)	20.0 (0.1)	20.0 (0.1)	20.3 (0.1)	20.4 (0.1)	20.4 (0.2)	19.9 (0.1)	20.3 (0.0)
2000/1 MCS	7	16.1 (0.1)	16.3 (0.0)	16.3 (0.1)	16.4 (0.1)	16.6 (0.1)	16.7 (0.2)	16.3 (0.0)	16.6 (0.0)
	11	18.3 (0.1)	18.7 (0.1)	19.0 (0.1)	19.1 (0.1)	19.6 (0.1)	19.5 (0.3)	18.7 (0.1)	19.4 (0.1)
	14	21.0 (0.2)	21.5 (0.1)	21.5 (0.1)	21.9 (0.1)	22.4 (0.2)	22.9 (0.4)	21.5 (0.1)	22.2 (0.1)
Weight									
Cohort	Age								
1946 NSHD	7	23.6 (0.2)	23.3 (0.1)	22.5 (0.2)	22.3 (0.1)	22.3 (0.1)	22.0 (0.3)	22.9 (0.1)	22.3 (0.1)
	11	36.2 (0.5)	36.0 (0.3)	35.1 (0.3)	34.6 (0.2)	34.7 (0.3)	33.9 (0.6)	35.2 (0.2)	34.8 (0.1)
	15	53.8 (0.7)	54.9 (0.4)	53.2 (0.4)	52.9 (0.3)	53.6 (0.4)	51.9 (0.8)	53.6 (0.3)	53.3 (0.2)
1958 NCDS	7	23.3 (0.2)	23.1 (0.1)	22.7 (0.1)	22.6 (0.1)	22.5 (0.1)	22.1 (0.1)	23.0 (0.1)	22.5 (0.0)
	11	35.6 (0.3)	35.9 (0.2)	35.2 (0.2)	34.9 (0.1)	34.7 (0.2)	34.0 (0.2)	35.3 (0.1)	35.0 (0.1)
	16	54.0 (0.4)	54.3 (0.2)	53.9 (0.3)	53.7 (0.2)	53.4 (0.3)	52.6 (0.4)	53.9 (0.2)	53.6 (0.1)
1970 BCS	10	36.1 (0.2)	36.1 (0.1)	36.0 (0.2)	35.7 (0.1)	35.6 (0.1)	35.1 (0.2)	36.2 (0.1)	35.6 (0.1)
	16	53.9 (0.5)	53.7 (0.2)	53.7 (0.4)	53.5 (0.2)	53.3 (0.4)	53.3 (0.6)	53.7 (0.2)	53.4 (0.2)
2000/1 MCS	7	24.4 (0.2)	24.4 (0.1)	24.5 (0.2)	24.4 (0.1)	24.7 (0.2)	25.0 (0.4)	24.4 (0.1)	24.6 (0.1)
	11	39.8 (0.4)	40.1 (0.2)	40.7 (0.3)	40.7 (0.2)	41.8 (0.3)	41.8 (0.8)	40.2 (0.1)	41.1 (0.2)
	14	60.0 (0.6)	60.6 (0.2)	60.7 (0.4)	61.1 (0.3)	62.3 (0.5)	63.9 (1.2)	60.7 (0.2)	61.8 (0.2)

Height		Father's social class						Maternal education	
Cohort	Age	I Professional	II	III Non-manual	III Manual	IV	V Unskilled	High	Low (compulsory)
1946 NSHD	7	121.9 (0.4)	120.9 (0.3)	120.3 (0.3)	118.9 (0.2)	118.8 (0.3)	117.4 (0.4)	120.7 (0.2)	118.8 (0.1)
	11	143.3 (0.5)	142.8 (0.3)	142.3 (0.4)	140.6 (0.2)	140.8 (0.3)	138.4 (0.5)	142.2 (0.3)	140.6 (0.2)
	15	163.9 (0.6)	164.2 (0.4)	163.5 (0.4)	161.8 (0.3)	162.1 (0.4)	159.3 (0.6)	163.5 (0.3)	161.9 (0.2)
1958 NCDS	7	122.4 (0.2)	121.9 (0.1)	121.5 (0.2)	120.5 (0.1)	120.2 (0.1)	119.3 (0.2)	121.8 (0.1)	120.4 (0.1)
	11	144.1 (0.3)	143.5 (0.2)	143.3 (0.2)	141.8 (0.1)	141.5 (0.2)	140.6 (0.2)	143.4 (0.1)	141.9 (0.1)
	16	163.6 (0.4)	162.8 (0.2)	162.7 (0.3)	161.3 (0.1)	160.7 (0.2)	160.3 (0.3)	162.8 (0.2)	161.1 (0.1)
1970 BCS	10	143.4 (0.3)	143.1 (0.1)	142.9 (0.2)	141.8 (0.1)	141.4 (0.2)	140.7 (0.2)	143.2 (0.1)	141.6 (0.1)
	16	162.6 (0.5)	162.4 (0.2)	162.3 (0.3)	160.7 (0.2)	160.1 (0.3)	160.3 (0.5)	162.5 (0.2)	160.6 (0.1)
2000/1 MCS	7	123.5 (0.3)	122.8 (0.1)	122.8 (0.2)	122.1 (0.1)	122.0 (0.2)	122.6 (0.4)	122.8 (0.1)	122.1 (0.1)
	11	146.9 (0.3)	145.9 (0.1)	145.9 (0.2)	145.3 (0.2)	145.4 (0.2)	146.0 (0.5)	146.0 (0.1)	145.2 (0.1)
	14	170.1 (0.4)	169.1 (0.2)	168.9 (0.3)	168.2 (0.2)	168.0 (0.3)	168.5 (0.7)	169.1 (0.1)	168.0 (0.1)

Supplementary Table 2. Social class, maternal education, and missing data tabulations in four British birth cohort studies

	1946 NSHD		1958 NCDS		1970 BCS		2000/1 MCS	
	N	%	N	%	N	%	N	%
Father's social class at 10/11y, N (%)								
I Professional	238	(4.4)	577	(3.4)	604	(3.5)	469	(2.9)
II Managerial and technical	766	(14.3)	2483	(14.4)	2984	(17.3)	3839	(23.4)
III Skilled non-manual	604	(11.3)	1315	(7.6)	1333	(7.7)	1198	(7.3)
III Skilled manual	1334	(24.9)	5305	(30.8)	5174	(29.9)	2034	(12.4)
IV Partly skilled	745	(13.9)	1925	(11.2)	1754	(10.1)	1292	(7.9)
V Unskilled	241	(4.5)	1113	(6.5)	872	(5.0)	256	(1.6)
Missing	1434	(26.7)	4484	(26.1)	4569	(26.4)	7316	(44.6)
Missing father's social class data Amongst those with valid BMI data at 10/11y	308	(7.82)	577	(4.90)	370	(3.19)	2176	(19.79)
Maternal Education (binary)[a], N (%)								
Post-compulsory	1237	(23.1)	4229	(24.6)	5624	(32.5)	8183	(49.9)
Compulsory schooling only	3105	(57.9)	12729	(74.0)	10741	(62.1)	8167	(49.8)
Missing	1020	(19.0)	244	(1.4)	925	(5.3)	54	(0.3)
Missing maternal education class data Amongst those with valid BMI data at 10/11y	231	(5.87)	140	(1.19)	387	(3.34)	13	(0.12)
Maternal education (number of years)[b], N (%)								
≥17	384	(7.2)	994	(5.8)	2906	(16.8)	8183	(49.9)
16	471	(8.8)	1170	(6.8)	2714	(15.7)	6254	(38.1)
15	382	(7.1)	3153	(18.3)	9542	(55.2)	1502	(9.2)
≤14	3105	(57.9)	4935	(28.7)	1166	(6.7)	411	(2.5)
Missing	1020	(19.0)	6950	(40.4)	962	(5.6)	54	(0.3)
Valid BMI data at 10/11y	3937	(73.4)	11770	(68.4)	11601	(67.1)	10996	(67.0)
Missing BMI data at 10/11y	1425	(26.6)	5432	(31.6)	5689	(32.9)	5408	(33.0)
Valid BMI data at 14/16y	3581	(66.8)	10236	(59.5)	7513	(43.5)	9389	(57.2)
Missing BMI data at 14/16y	1781	(33.2)	6966	(40.5)	9777	(56.5)	7015	(42.8)

[a]Measured at birth in 1958 NCDS and 1970 BCS, 9 months in 2001 MCS, and age 6 in 1946 NSHD; [b]measured at the same ages as binary maternal education, except at age 16 years in 1958 NCDS. Note: missing data includes death/emigration from Britain/drop out/refusal to participate.

Supplementary Table 3. Odds of missing data at 14/16 years according to social class and preceding BMI in four British birth cohort studies

Cohort	N	Social class (slope index inequality) OR (95% CI)	BMI at 10/11 years OR (95% CI)
1946 NSHD	3,629	1.08 (0.72, 1.61)	1.00 (0.96, 1.05)
1958 NCDS	11,193	1.37 (1.18, 1.60)	1.03 (1.01, 1.04)
1970 BCS70	11,231	1.93 (1.68, 2.21)	1.00 (0.99, 1.03)
2001 MCS	8,820	2.29 (1.82, 2.88)	1.06 (1.04, 1.08)

Supplementary Table 4. Father's social class and BMI, weight and height in childhood/adolescence (7-15 years): estimates (SE) from separate multilevel models, scaled to show estimated BMI differences at 11 years

	Cohort (age) 1946 NSHD (7, 11, 15)	P	1958 NCDS (7, 11, 15)	P	1970 BCS (10, 15)	P	20001 MCS (7, 11, 15)	P
Body mass index, kg/m^2								
Number of observations	10401		30667		17880		24553	
β Age	0.56 (0.01)	<0.001	0.53 (0.0065)	<0.001	0.63 (0.019)	<0.001	0.64 (0.0081)	<0.001
β SEP ridit score	0.12 (0.12)	0.333	0.1 (0.072)	0.154	0.14 (0.085)	0.093	0.96 (0.13)	<0.001
SEP*age interaction	0.026 (0.018)	0.159	0.033 (0.012)	0.004	0.12 (0.035)	0.001	0.1 (0.015)	<0.001
β Gender (ref female)	0.17 (0.053)	0.001	0.0082 (0.034)	0.808	-0.39 (0.037)	<0.001	0.12 (0.051)	0.023
Constant	18 (0.072)		18 (0.044)		18 (0.052)		19 (0.079)	
Random effect: sd(age)	0.31 (0.0062)		0.17 (0.0047)				0.35 (0.013)	
Random effect: sd(_cons)	2.6 (0.059)		0.36 (0.24)				2.1 (0.19)	
Random effect: sd(Residual)	0.77 (0.015)		1.4 (0.011)				1.2 (-0.096)	
Weight, kg								
β Age	3.9 (0.031)	<0.001	3.8 (0.022)	<0.001	4.4 (0.057)	<0.001	4.6 (0.03)	<0.001
β SEP ridit score	-1.6 (0.35)	<0.001	-1.5 (0.21)	<0.001	-1 (0.25)	<0.001	1 (0.36)	0.003
SEP*age interaction	-0.027 (0.057)	0.638	-0.05 (0.038)	0.191	0.11 (0.1)	0.274	0.18 (0.054)	0.001
β Gender (ref female)	0.29 (0.12)	0.011	-0.66 (0.082)	<0.001	2.1 (0.11)	<0.001	-0.37 (0.1)	<0.001
Constant	38 (0.2)		38 (0.13)		35 (0.15)		42 (0.21)	
Random effect: sd(age)	0.97 (0.017)		0.78 (0.009)				1.2 (0.022)	
Random effect: sd(_cons)	6.2 (0.14)		3 (0.085)				5.6 (0.28)	
Random effect: sd(Residual)	2.3 (0.033)		3.7 (-0.019)				3.3 (-0.13)	
Height, cm								
β Age	5.4 (0.024)	<0.001	5.1 (0.017)	<0.001	4.9 (0.056)	<0.001	5.8 (0.017)	<0.001
β SEP ridit score	-4 (0.36)	<0.001	-3.3 (0.21)	<0.001	-2.9 (0.24)	<0.001	-1.4 (0.26)	<0.001
SEP*age interaction	-0.00115 (0.044)	0.997	-0.032 (0.03)	0.286	-0.066 (0.1)	0.509	-0.092 (0.031)	0.002
β Gender (ref female)	0.32 (0.19)	0.085	0.13 (0.11)	0.231	4.2 (0.11)	<0.001	-0.3 (0.13)	0.022
Constant	143 (0.22)		143 (0.13)		141 (0.15)		146 (0.18)	
Random effect: sd(age)	0.71 (0.013)		0.4 (0.013)				0.54 (0.014)	
Random effect: sd(_cons)	7.6 (0.16)		3.8 (0.19)				5 (0.19)	
Random effect: sd(Residual)	1.9 (0.036)		3.7 (0.027)				2.6 (-0.062)	

Supplementary Table 5. Father's social class (slope index of inequality) and risk of thinness, overweight or obesity

Age, cohort	BMI category	N	Difference in predicted probability	95% CI	Relative risk ratio	95% CI
7 years						
1946 NSHD	Thinness	296	0.022	(-0.018, 0.062)	1.37	(0.78, 2.41)
	Normal	2964	-0.017	(-0.072, 0.037)	Ref	
	Overweight	237	-0.022	(-0.059, 0.015)	0.73	(0.40, 1.35)
	Obese	23	0.017	(-0.002, 0.036)	8.07	(1.06, 61.73)
1958 NCDS	Thinness	1146	0.002	(-0.020, 0.023)	1.02	(0.81, 1.27)
	Normal	8537	0.000	(-0.027, 0.028)	Ref	
	Overweight	780	-0.003	(-0.021, 0.015)	0.96	(0.74, 1.26)
	Obese	187	0.001	(-0.008, 0.010)	1.05	(0.62, 1.78)
2001 MCS	Thinness	632	0.007	(-0.016, 0.030)	1.25	(0.87, 1.80)
	Normal	6127	-0.093	(-0.136, -0.049)	Ref	
	Overweight	1120	0.043	(0.011, 0.076)	1.57	(1.17, 2.11)
	Obese	461	0.043	(0.024, 0.062)	2.71	(1.80, 4.08)
11 years						
1946 NSHD	Thinness	388	0.021	(-0.021, 0.063)	1.26	(0.78, 2.03)
	Normal	2921	-0.015	(-0.070, 0.040)	Ref	
	Overweight	275	-0.023	(-0.059, 0.012)	0.73	(0.42, 1.28)
	Obese	45	0.017	(-0.004, 0.038)	3.19	(0.84, 12.09)
1958 NCDS	Thinness	1668	0.004	(-0.020, 0.028)	1.04	(0.86, 1.25)
	Normal	8469	-0.008	(-0.037, 0.021)	Ref	
	Overweight	880	0.003	(-0.015, 0.021)	1.05	(0.81, 1.35)
	Obese	176	0.001	(-0.007, 0.010)	1.10	(0.64, 1.90)
1970 BCS	Thinness	1000	0.002	(-0.017, 0.021)	1.06	(0.84, 1.34)
	Normal	9327	-0.026	(-0.051, -0.001)	Ref	
	Overweight	897	0.021	(0.003, 0.039)	1.35	(1.05, 1.74)
	Obese	25	0.003	(-0.000, 0.007)	4.07	(0.93, 17.84)
2001 MCS	Thinness	612	-0.018	(-0.039, 0.003)	0.90	(0.63, 1.27)
	Normal	5875	-0.115	(-0.156, -0.073)	Ref	
	Overweight	1812	0.073	(0.037, 0.110)	1.71	(1.35, 2.17)
	Obese	521	0.059	(0.041, 0.078)	3.73	(2.51, 5.54)
15 years						
1946 NSHD	Thinness	280	0.027	(-0.012, 0.067)	1.53	(0.93, 2.52)
	Normal	2645	-0.082	(-0.143, -0.022)	Ref	
	Overweight	296	0.043	(-0.004, 0.090)	1.76	(1.02, 3.05)
	Obese	41	0.012	(-0.009, 0.033)	2.66	(0.58, 12.29)
1958 NCDS	Thinness	920	-0.005	(-0.028, 0.018)	0.99	(0.77, 1.27)
	Normal	7035	-0.028	(-0.058, 0.003)	Ref	
	Overweight	736	0.021	(-0.000, 0.041)	1.33	(1.01, 1.75)
	Obese	133	0.012	(0.002, 0.022)	2.30	(1.22, 4.31)
1970 BCS	Thinness	857	0.006	(-0.023, 0.035)	1.14	(0.88, 1.48)
	Normal	5077	-0.060	(-0.097, -0.024)	Ref	
	Overweight	606	0.039	(0.014, 0.064)	1.66	(1.22, 2.25)
	Obese	109	0.015	(0.004, 0.027)	2.74	(1.37, 5.45)
2001 MCS	Thinness	467	-0.022	(-0.041, -0.003)	0.79	(0.56, 1.11)
	Normal	5067	-0.093	(-0.135, -0.051)	Ref	
	Overweight	1375	0.049	(0.012, 0.085)	1.51	(1.16, 1.96)
	Obese	484	0.067	(0.044, 0.089)	3.50	(2.30, 5.32)

Supplementary Table 6. Body mass index (BMI), weight and height during childhood/adolescence: averages and socioeconomic differences

Cohort	Age	Year	n	BMI, kg/m^2 Mean	Median	Social class difference, SII (95% CI)	Weight, kg Mean	Median	Social class difference, SII (95% CI)	Height, cm Mean	Median	Social class difference, SII (95% CI)
1946 NSHD	7	1953	3783	15.8	15.6	0.2 (0.0, 0.5)	22.7	22.2	-1.1 (-1.6, -0.6)	119.8	119.4	-3.8 (-4.7, -2.9)
1958 NCDS	7	1964	12565	15.7	15.5	0.1 (-0.1, 0.2)	22.7	22.2	-1.0 (-1.3, -0.7)	120.8	120.6	-2.8 (-3.2, -2.3)
2001 MCS	7	2008	11645	16.4	16.0	0.6 (0.4, 0.7)	24.5	23.7	0.3 (-0.2, 0.7)	122.6	122.4	-1.5 (-1.9, -1.0)
1946 NSHD	11	1957	3706	17.4	17.0	0.3 (-0.1, 0.7)	35.2	33.7	-0.9 (-2.0, 0.2)	141.6	140.6	-3.2 (-4.4, -2.0)
1958 NCDS	11	1967	11630	17.3	16.7	0.4 (0.2, 0.6)	35.1	33.6	-0.7 (-1.3, -0.1)	142.3	142.2	-3.1 (-3.7, -2.5)
1970 BCS	10	1980	11214	17.4	17.1	0.2 (0.0, 0.4)	35.8	35.0	-1.0 (-1.4, -0.6)	142.2	142.0	-3.0 (-3.5, -2.5)
2001 MCS	11	2012	10983	18.9	18.2	1.3 (1.0, 1.6)	40.5	38.9	1.8 (0.9, 2.6)	145.7	145.5	-1.8 (-2.5, -1.2)
1946 NSHD	14	1961	3359	20.4	20.0	0.5 (0.1, 1.0)	53.6	52.5	-0.6 (-2.2, 1.0)	162.9	162.2	-3.2 (-4.5, -1.8)
1958 NCDS	16	1974	10074	20.2	19.7	0.6 (0.4, 0.9)	53.7	52.5	-0.7 (-1.5, 0.1)	161.7	161.3	-3.5 (-4.1, -2.9)
1970 BCS	16	1986	7264	20.2	19.7	0.7 (0.4, 1.0)	53.5	52.3	-0.4 (-1.3, 0.6)	161.4	161.3	-3.2 (-3.9, -2.5)
2001 MCS	14	2015	9381	21.7	20.9	1.4 (1.0, 1.8)	60.9	59.2	2.3 (1.1, 3.6)	168.9	168.5	-2.1 (-2.8, -1.4)

Notes: means and SII (slope index of inequality) are gender-adjusted. Maternal education defined using binary measures.

a) Body mass index

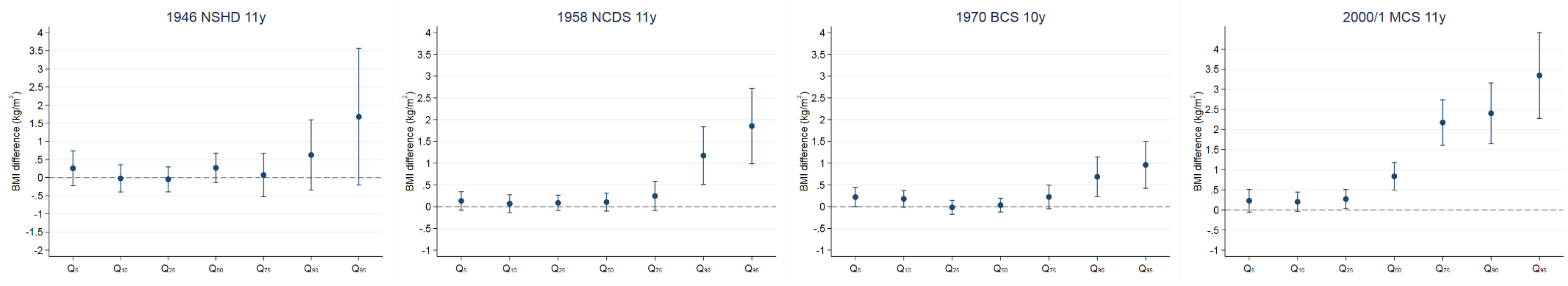

b) Weight

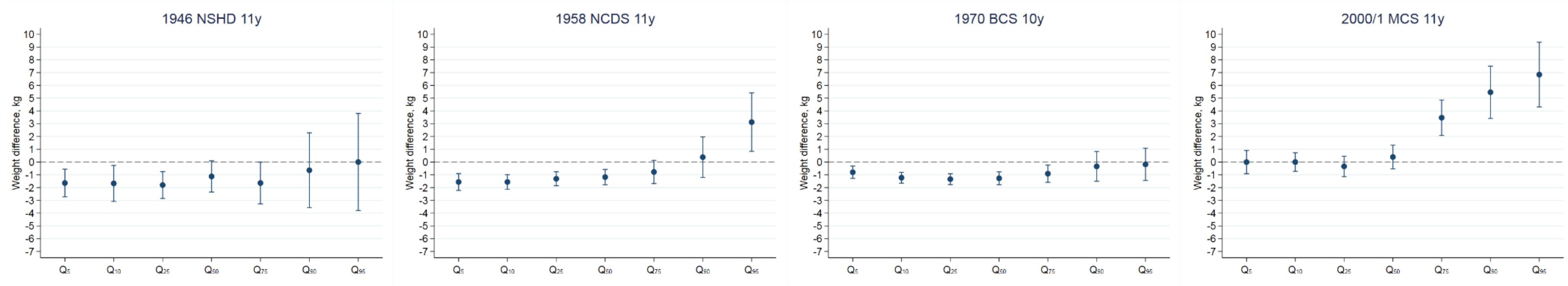

c) Height

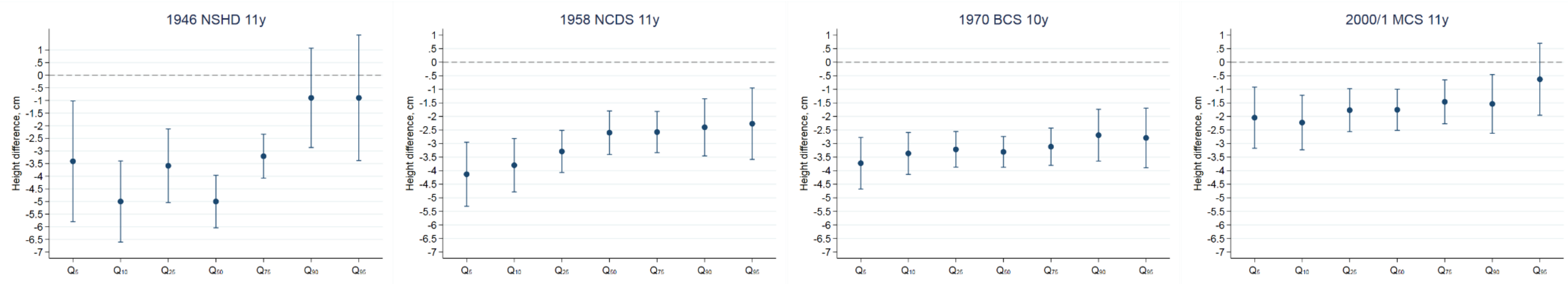

Supplementary Figure 1. Estimated differences in BMI, weight, and height (age 10/11 years) in the lowest verses highest maternal education (slope index of inequality): quantile regression estimates (95% CI).

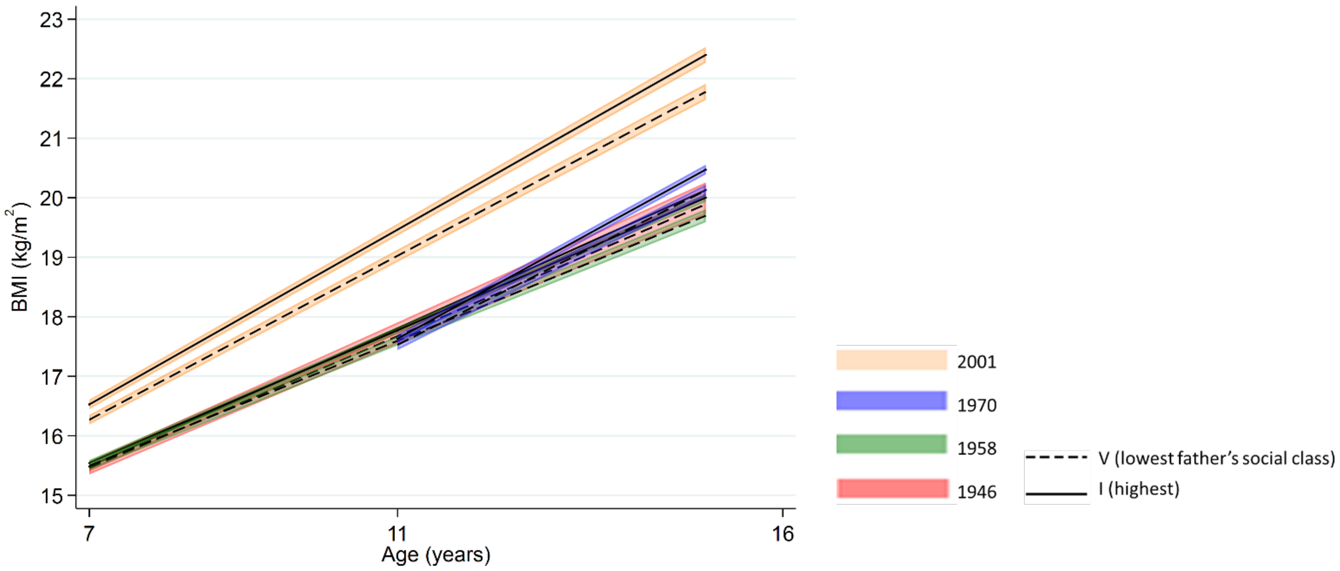

Supplementary Figure 2. Body mass index (BMI) across childhood and adolescence in relation to maternal education attainment in the 1946, 1958, 1970, and 2001 British birth cohort studies. Note: lines show estimated BMI along with 95% confidence intervals at each age among women, estimated using multilevel general linear regression models.

a) Body mass index

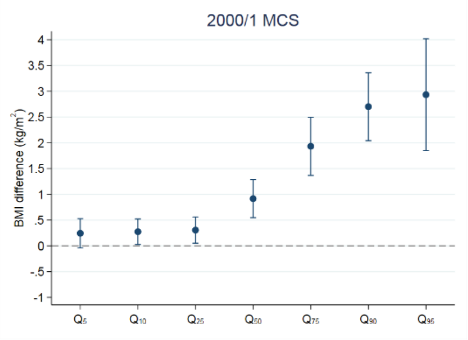

b) Weight

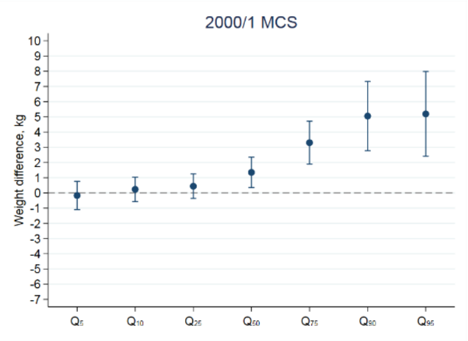

c) Height

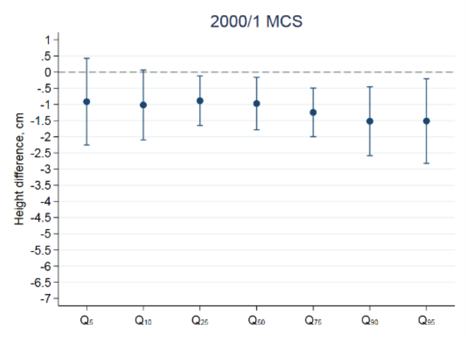

Supplementary Figure 3. Estimated differences in BMI, weight and height (age 11 years) in the lowest verses highest fathers social class (slope index of inequality): quantile regression estimates (95% CI) in the 2001 MCS (restricted to white participants)
